# Risk Factors and Comparative Epidemiology of Pediculosis Capitis Among Schoolchildren in Baghlan Province, Afghanistan: A Public Health Framework for Prevention and Control

**DOI:** 10.1002/hsr2.73419

**Published:** 2026-11-09

**Authors:** Mohammad Musawer Sharifi, Sayed Hussain Mosawi, Ahmad Jamshid Mehrpoor, Reza Fotouhi‐Ardakani, Shah Zaman Shirzad, Abdullah Sahar, Abdul Wakil Qarluq, Mohammad Esmail Ahmadyar, Atefeh Khoshkchali, Hadia Azami, Aida Vafae Eslahi, Morteza Habibi, Milad Badri

**Affiliations:** ^1^ Medical Sciences Research Center Ghalib University Kabul Afghanistan; ^2^ Cellular and Molecular Research Center Qom University of Medical Sciences Qom Iran; ^3^ Department of Paraclinic, Faculty of Medicine Spinghar University Kabul Afghanistan; ^4^ Department of Microbiology, Faculty of Medicine Afghan International Islamic University Kabul Afghanistan; ^5^ Non‐communicable Diseases Research Center, Research Institute for Prevention of Non‐communicable Diseases Qazvin University of Medical Sciences Qazvin Iran; ^6^ Safety and Health Research Center Qazvin University of Medical Sciences Qazvin Iran

**Keywords:** Afghanistan, head lice, prevalence, public health, risk factors, schoolchildren

## Abstract

**Background and Aims:**

Pediculosis capitis, caused by *Pediculus humanus capitis*, remains a significant public health concern among school‐aged children worldwide, particularly in resource‐limited settings. This study aimed to determine the prevalence, associated risk factors, and epidemiological characteristics of head lice infestation among primary schoolchildren in Baghlan Province, Afghanistan, to inform evidence‐based prevention and control strategies.

**Methods:**

A descriptive‐analytical cross‐sectional study was conducted from January 2024 to December 2024 among 507 schoolchildren from primary schools across Baghlan Province, Afghanistan. Scalp examinations were performed under flashlight to detect adult lice, nymphs, and viable nits. A structured questionnaire collected data on demographic, socioeconomic, behavioral, and household‐related factors. Univariable and multivariable logistic regression analyses were performed to identify independent risk factors associated with pediculosis capitis.

**Results:**

The overall prevalence was 6.9% (35/507). Family history of infestation was reported by 65.7%. In univariable analysis, hairbrush sharing (OR = 2.17, 95% CI: 1.05–4.40; *p* = 0.035) and bedroom sharing (OR = 1.83, 95% CI: 1.01–3.65; *p* = 0.048) were significantly associated with infestation. However, none of the variables present in the sensitivity analysis model showed a statistically significant relationship with the outcome (*p* > 0.05).

**Conclusion:**

Pediculosis capitis remains a persistent public health concern among Afghan schoolchildren. The multifactorial nature of infestation underscores the need for comprehensive, school‐based interventions integrating routine screening, health education, early case detection, and timely treatment, alongside household‐level hygiene promotion. Future adequately powered multicenter studies are warranted to further elucidate infestation determinants across different regions of Afghanistan.

## 1 Introduction

The condition of pediculosis capitis involves infestations of the head and hair by *Pediculus humanus capitis*, an obligate ectoparasite that causes one of the most common and widespread skin infections by parasites in the world today, with millions of people affected yearly regardless of social class [1, 2]. This wingless and blood‐feeding insect that is exclusively specific to humans throughout its complete life cycle stays on the scalp and feeds solely on human blood [3, 4, 5]. The adult female louse reproduces up to ten eggs daily, attaching them firmly to the base of the hair shaft using a chitinous material, and requires the warmth of the scalp to incubate the eggs [6, 7]. These eggs hatch after six to ten days into nymphs that develop into adults within 1 week, thus forming an infestation life cycle that is transferred mainly from person to person, especially among children [8].

Pediculosis Capitis is nonetheless very significant from the viewpoint of public health owing to its prevalence, morbidity, and psychological implications [9, 10]. There has been found to be very high prevalence of the infestation worldwide, with recent meta‐analysis conducted indicating that the prevalence rate in the world population is 19% among school children [11]. The primary symptom that occurs due to the infestation is pruritus that results from hypersensitivity reaction to the saliva of the louse, leading to excoriations, insomnia, and even secondary infection such as impetigo [12, 13]. Furthermore, chronic and severe infestations, especially in impoverished communities, can lead to iron deficiency anemia [14]. Beyond these physical manifestations, pediculosis capitis also has a large social stigma associated with it which causes embarrassment, psychological distress, and anxiety for the child and his or her family, and even leads to social isolation and school absenteeism [10, 15, 16]. The economic burden related to therapy, parent's missed work days, and unwarranted school exclusion is also considerable, and thus emphasizing the importance of this condition as a public health issue [17].

Diagnosis of pediculosis is the key to successful treatment, which requires more than just physical inspection; in fact, it involves detection of a live louse, using a fine detection comb, and wet‐combing is found to be four times more efficient than physical inspection [18, 19]. This is very important, since the mere occurrence of empty eggshells (nits) is not enough to imply the existence of an infestation [3, 20]. There have been many changes in the treatment of pediculosis capitis during the past decades. Although the use of neurotoxic topical pediculicides, like permethrin and pyrethrins, has always been the key strategy in pediculosis capitis management, their efficacy has declined as resistance has become common [21, 22]. Therefore, the modern management of the patient makes use of a multi‐dimensional strategy that includes physical methods such as combing and also the use of new topical drugs that operate through a mode of action that is not based on neurotoxicity and includes Ivermectin, Spinosad, Benzyl Alcohol, and Dimethicone [23, 24, 25]. In addition, for resistant cases, oral ivermectin or trimethoprim‐sulfamethoxazole may be administered [26, 27]. Proper management with the scientific method is particularly essential in regions where there is a lack of adequate health care facilities and the problem is prevalent [6, 28]. The objective of this research is to conduct an investigation regarding the prevalence, risk factors, and existing management of pediculosis capitis in Baghlan Province, Afghanistan, as an attempt to provide vital information that would be used in the formulation of policies and strategies to combat this neglected parasitic infection.

## 2 Methods

### 2.1 Study Location, Design and Sample

This descriptive‐analytical cross‐sectional study was conducted over a 1‐year period from January 2024 to December 2024 among school children in Baghlan province, Afghanistan. Twenty‐five schools were selected for the study, comprising seven girls' schools and eighteen boys’ schools. Students in grades 1–6 were eligible for participation, with the individual student serving as the sampling unit. Participants were randomly selected, with each student having an equal probability of selection and the selection of individual made independently of the selection of others. The schools served as settings for participant recruitment rather than as sampling clusters. Overall, 507 students (277 males and 230 females) were enrolled. Each participant underwent an individual, private scalp examination under a flashlight to detect all stages of head lice infestation, including adult lice, nymphs, and viable nits; the presence of any of these stages was considered a positive case. Prior to the physical examination, a structured questionnaire was administered to collect data on potential risk factors, including school grade, hair characteristics (length, texture, color, thickness), frequency and method of hair washing (soap, shampoo, or both), sharing practices of hair‑related items (combs, scarves, towels, pillows), parental education and occupation, family income, family size, number of persons sharing a bedroom, history of previous infestation, frequency of hair salon visits, hair combing habits, access to healthcare services, presence of a school health coach, and self‑reported scalp sweating. Univariable and multivariable logistic regression analyses were performed to identify independent risk factors associated with pediculosis capitis (Figures 1 and 2).

**Figure 1:**
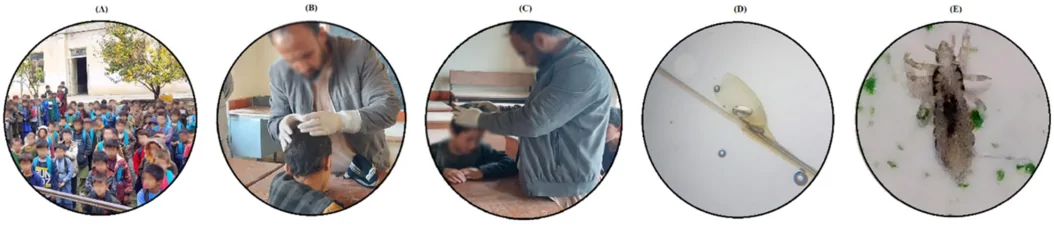
(A–C) Scalp screening samples obtained from schoolchildren during routine examination. (D) Representative image of a nit (lice egg) attached to a hair shaft. (E) Adult stage of the head lice (Pediculus humanus capitis), showing characteristic morphology.

**Figure 2:**
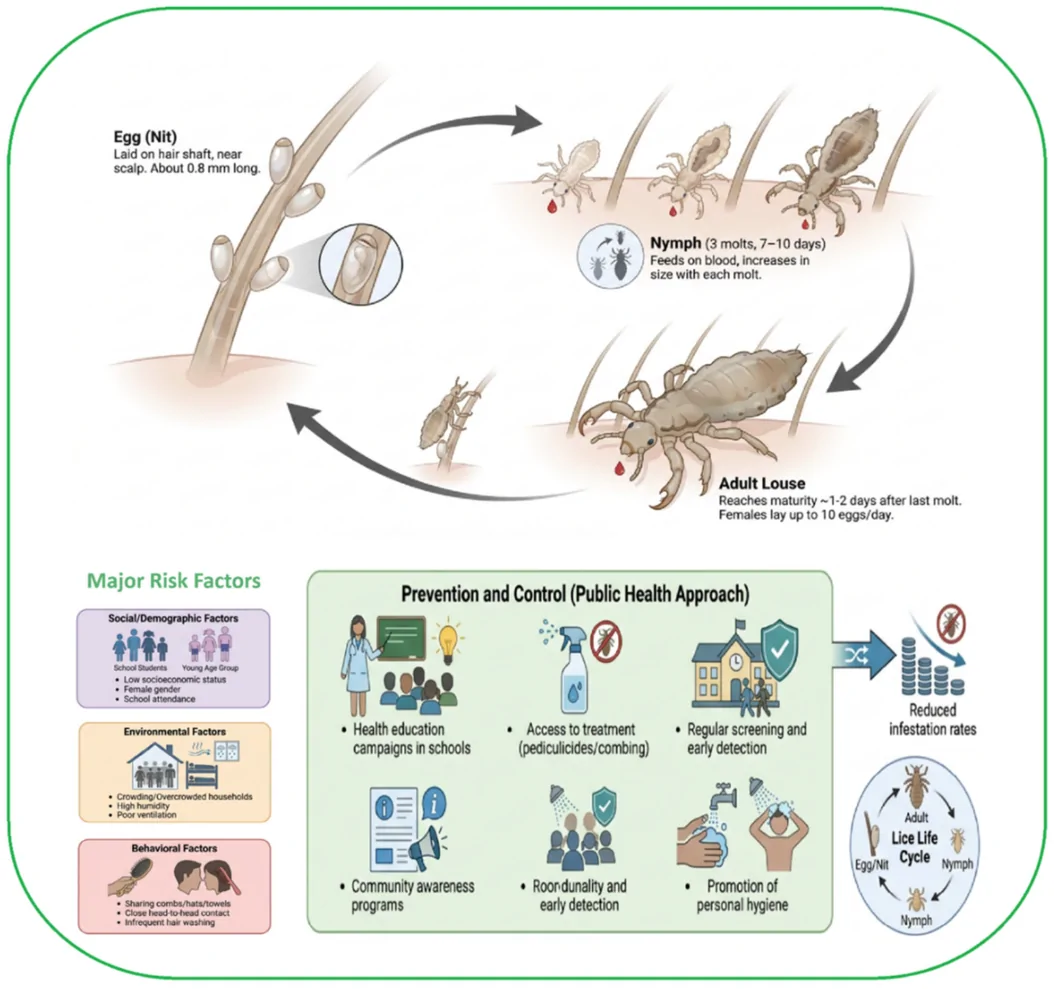
Life cycle of head lice (Pediculus humanus capitis), from egg (nit) through nymphal stages (7–10 days, 3 molts) to blood‐feeding adults, alongside major risk factors (social/demographic, environmental, behavioral) and a public health prevention and control strategy (education, treatment access, screening, hygiene promotion) aimed at reducing infestation rates. This framework was designed with the assistance of an AI diagramming tool (Figure Labs, https://chat.figurelabs.ai/chat).

### 2.2 Statistical Analysis

Data were analyzed using STATA software (version 17; StataCorp, USA). Categorical variables were summarized as frequencies and percentages. Associations between participant characteristics and pediculosis capitis were initially evaluated using univariable logistic regression.

To examine the effect of other variables affecting the outcome, a sensitivity analysis was performed with a significance level of less than 0.2. Adjusted odds ratios (AORs) and corresponding 95% confidence intervals (CIs) were estimated. There were no missing data for variables included in the regression analyses. Statistical significance was defined as a two‐sided *p* < 0.05 in final model.

## 3 Results

### 3.1 Participant Characteristics

A total of 507 schoolchildren participated in a study aimed at investigating the epidemiology of pediculosis in Baghlan province (Figure 3). Pediculosis capitis was identified in 35 children, corresponding to an overall prevalence of 6.9%. Participant characteristics according to pediculosis status are presented in Table 1.

**Figure 3:**
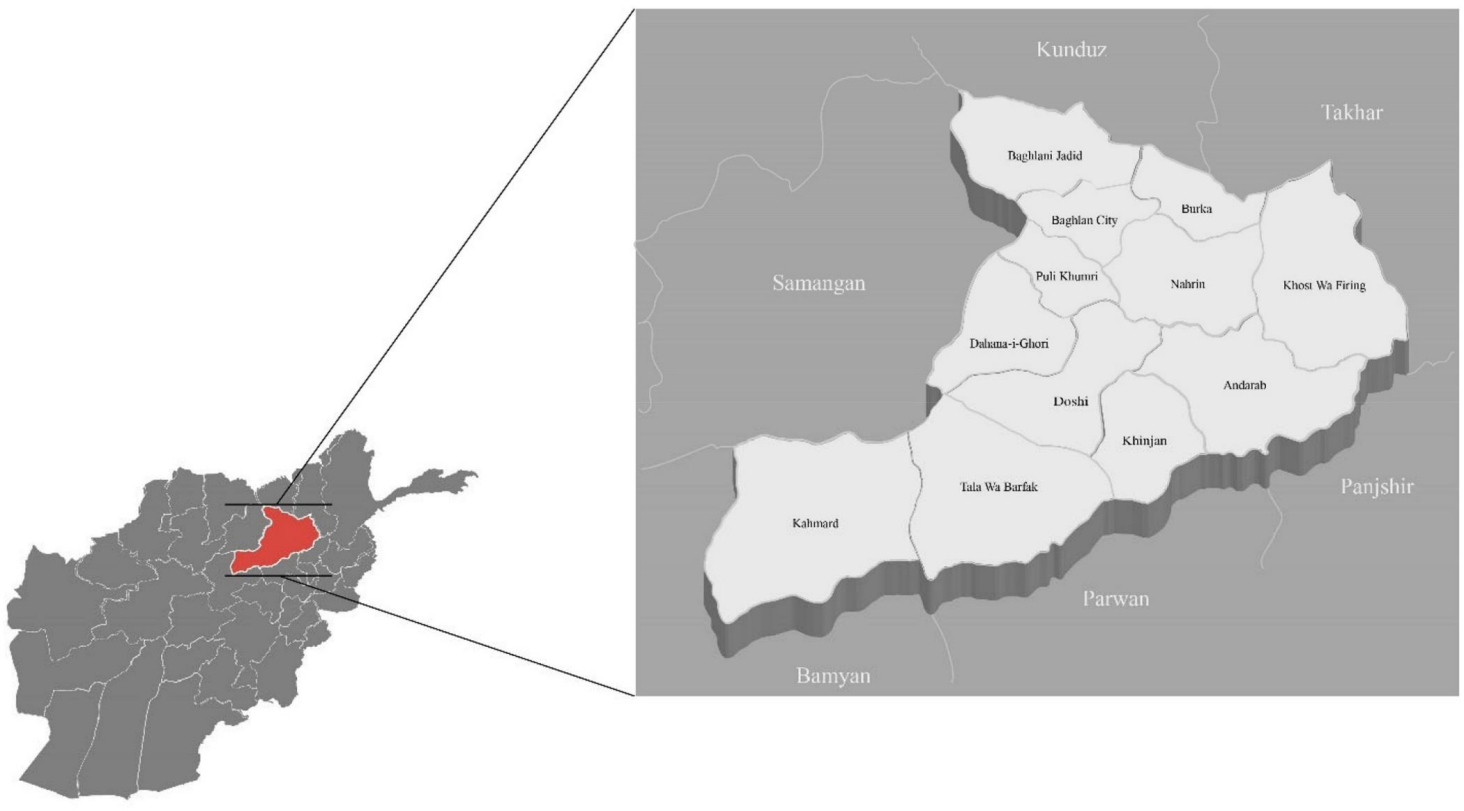
The study was carried out in Baghlan Province, Afghanistan, and the study sample comprised schoolchildren selected from across the province.

**Table 1:** Participant characteristics according to pediculosis capitis status.

Characteristic	Category	No pediculosis *n* (%)	Pediculosis *n* (%)
Sex	Male	255 (54)	22 (62.9)
	Female	217 (46)	13 (37.1)
Grade	1–2	293 (62.2)	22 (62.9)
	3–5	179 (37.8)	13 (37.1)
Father's education	Illiterate/Primary	250 (52.9)	18 (51.4)
	Secondary/Higher	222 (47.1)	17 (48.6)
Mother's education	Illiterate/Primary	253 (53.6)	15 (42.9)
	Secondary/Higher	219 (46.4)	20 (57.1)
Economic status	Poor	209 (44.3)	18 (51.4)
	Middle/Good	263 (55.7)	17 (48.6)
Family size	≤ 5	259 (54.9)	16 (45.7)
	> 5	213 (45.1)	19 (54.3)
Hairbrush sharing	No	371 (78.6)	22 (62.9)
	Yes	101 (21.4)	13 (37.1)
Clothes sharing	No	131 (27.8)	14 (40)
	Yes	341 (72.2)	21 (60)
Bedroom sharing	No	335 (70.9)	20 (57.1)
	Yes	137 (29.1)	15 (42.9)
Frequent scalp sweating	No	180 (38)	16 (45.7)
	Yes	292 (62)	19 (54.3)
Health coach in school	No	299 (63.4)	19 (54.3)
	Yes	173 (36.6)	16 (45.7)
Family history	No	207 (43.8)	12 (34.3)
	Yes	265 (56.1)	23 (65.7)
Access to healthcare	No	250 (53.1)	16 (45.7)
	Yes	222 (46.9)	19 (54.3)
Hair texture	Straight	261 (55.3)	19 (54.3)
	Non‐straight	211 (44.7)	16 (45.7)
Hair length	Short/Medium	357 (75.6)	27 (77.1)
	Long	115 (24.4)	8 (22.9)
Hair color	Brown/Black	338 (71.6)	22 (62.9)
	Blonde/Gray	134 (28.4)	13 (37.1)
Hair thickness	Thin	219 (46.4)	15 (42.9)
	Moderate	126 (26.6)	7 (20.0)
	Thick	127 (27.0)	13 (37.1)
Hair washing method	Soap	114 (24.1)	10 (28.6)
	Shampoo	212 (45.0)	17 (48.6)
	Both	146 (30.9)	8 (22.9)
Bathing frequency	≤ 2/week	338 (71.6)	24 (68.6)
	≥ 3/week	134 (28.4)	11 (31.4)
Hair salon visits	≤ 1/month	175 (37.1)	9 (25.7)
	≥ 2/month	297 (62.9)	26 (74.3)
Hair combing	Frequent	365 (77.3)	25 (71.4)
	Infrequent	107 (22.7)	10 (28.6)

*Note:* Among children with pediculosis, 62.9% were male and 62.9% were enrolled in grades 1–2. More than half belonged to households with poor economic status (51.4%) and larger family sizes (> 5 members; 54.3%). A family history of pediculosis was reported by 65.7% of affected children. Sharing a bedroom (42.9%) and sharing hairbrushes (37.1%) were also more common among children with pediculosis.

### 3.2 Univariable Analysis

Results of the univariable logistic regression analyses are presented in Table 2. Sharing hairbrushes with family members, and sharing bedroom with family members were the variables significantly associated with pediculosis capitis. Children who shared hairbrushes had more than twice the odds of infestation compared with those who did not (OR = 2.17, 95% CI: 1.05–4.40; *p* = 0.035). Also, children who share bedroom with others had 83% higher odds of infestation than who did not, which was not statistically significant relationship (OR = 1.83, 95% CI = 1.01–3.65; *p* = 0.089) (Table 2).

**Table 2:** Univariable logistic regression analysis.

Variable	Category	cOR (95% CI)	*p* value
Sex	Male	Ref	—
	Female	0.70 (0.34–1.40)	0.313
Grade	1–2	Ref	—
	3–5	0.97 (0.49–1.96)	0.935
Father's education	Illiterate/Primary	Ref	—
	Secondary/Higher	1.06 (0.53–2.10)	0.860
Mother's education	Illiterate/Primary	Ref	—
	Secondary/Higher	1.54 (0.78–3.08)	0.222
Economic status	Poor	Ref	—
	Middle/Good	0.75 (0.38–1.49)	0.413
Family size	≤ 5	Ref	—
	> 5	1.44 (0.72–2.87)	0.301
Hairbrush sharing	No	Ref	—
	Yes	2.17 (1.05–4.40)	0.035*
Clothes sharing	No	Ref	—
	Yes	0.57 (0.29–1.17)	0.124
Bedroom sharing	No	Ref	—
	Yes	1.83 (0.91–3.68)	0.089
Frequent scalp sweating	No	Ref	—
	Yes	0.73 (0.37–1.45)	0.358
Health coach in school	No	Ref	—
	Yes	1.45 (0.72–2.90)	0.287
Family history	No	Ref	—
	Yes	1.49 (0.72–3.07)	0.273
Access to healthcare	No	Ref	—
	Yes	1.33 (0.67–2.66)	0.408
Hair texture	Straight	Ref	—
	Non‐straight	1.04 (0.52–2.06)	0.905
Hair length	Short/Medium	Ref	—
	Long	0.92 (0.41–2.04)	0.841
Hair color	Brown/Black	Ref	—
	Blonde/Gray	1.49 (0.73–3.01)	0.273
Hair thickness	Thin	Ref	—
	Moderate	0.81 (0.32–2.04)	0.657
	Thick	1.49 (0.68–3.24)	0.309
Hair washing method	Soap	Ref	—
	Shampoo	0.91 (0.41–2.06)	0.829
	Both	0.62 (0.24–1.63)	0.337
Bathing frequency	≤ 2/week	Ref	—
	≥ 3/week	1.15 (0.55–2.42)	0.701
Hair salon visits	≤ 1/month	Ref	—
	≥ 2/month	1.7 (0.79–3.73)	0.182
Hair combing	Frequent	Ref	—
	Infrequent	1.36 (0.63–2.93)	0.426

Abbreviations: cOR, crude odds ratio; CI, confidence interval; Ref, reference category.

*p < 0.05.

Several other variables showed elevated odds of pediculosis capitis, although other factors did not reach statistical significance because their confidence intervals included the null value. These factors included family history of pediculosis (OR = 1.49, 95% CI: 0.72–3.07), frequent visits to a hair salon (OR = 1.7, 95% CI: 0.79–3.73), and thick hair compared with thin hair (OR = 1.49, 95% CI: 0.68–3.24). No statistically significant associations were observed for sex, grade, father education, economic status, family size, frequent scalp sweating, availability of a school health coach, access to healthcare, hair texture, hair length, hair color, hair washing method, bathing frequency, or hair‐combing frequency (Table 2).

### 3.3 Sensitivity Analysis

To assess the robustness of univariate regression findings and evaluate the effect of other variables affecting the outcome, a sensitivity analysis was conducted using a more liberal entry threshold of *p* < 0.2. The result demonstrated that none of the variables present in the model showed a statistically significant relationship with the outcome (*p* < 0.05) (Table 3).

**Table 3:** Sensitivity analysis.

Variable	Category	AOR (95% CI)	*p*‐value
Mother's education	Illiterate/Primary	Ref	—
	Secondary/Higher	1.58 (0.78–3.20)	0.195
Hairbrush sharing	No	Ref	—
	Yes	1.93 (0.64–5.75)	0.236
Clothes sharing	No	Ref	—
	Yes	1.11 (0.34–3.55)	0.864
Bedroom sharing	No	Ref	—
	Yes	1.41 (0.52–3.77)	0.495
Hair salon visits	No	Ref	—
	Yes	1.65 (0.75–3.63)	0.210

Abbreviations: AOR, adjusted odds ratio; CI, confidence interval; Ref, reference category.

Nevertheless, the adjusted odds ratios remained above 1.0 for all of factors in final model included; mother's education (aOR = 1.58, 95% CI: 0.78–3.20), bedroom sharing (aOR = 1.41, 95% CI: 0.52–3.77), and hairbrush sharing (aOR = 1.93, 95% CI: 0.64–5.75) (Table 3). Confidence intervals were relatively wide, reflecting the limited statistical precision resulting from the small number of outcome events (Figure 4).

**Figure 4:**
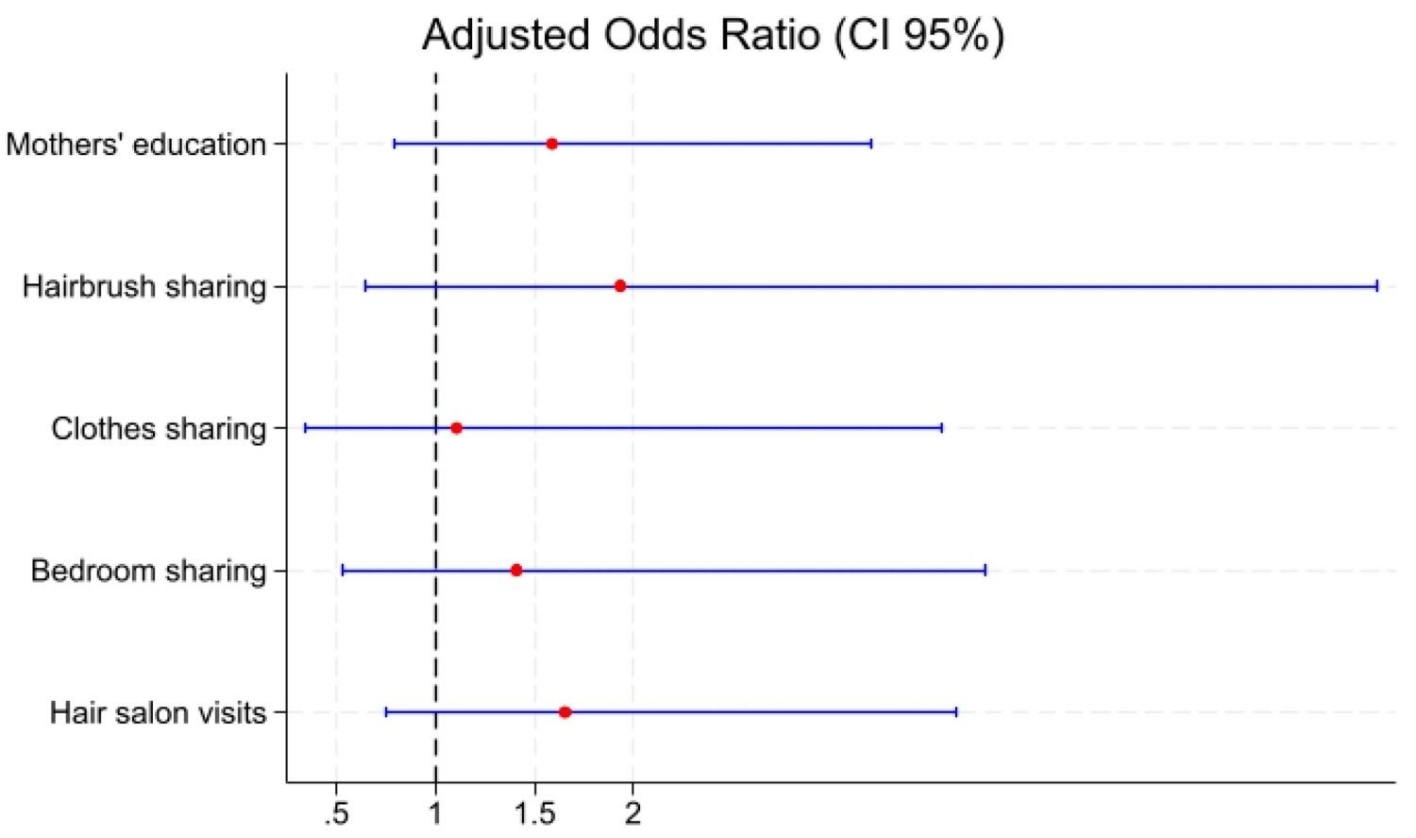
Adjusted odds ratio for pediculosis capitis by related factors.

## 4 Discussion

Pediculosis capitis remains a common parasitic infestation among school‐aged children and continues to represent an important public health concern worldwide. Although head lice infestation is not associated with severe systemic disease in most cases, it may cause persistent itching, scalp irritation, secondary bacterial infection, psychological distress, social stigma, and school absenteeism, thereby affecting children's quality of life and educational performance [3, 29]. The present study assessed a wide range of demographic, socioeconomic, behavioral, and household‐related variables as potential risk factors for pediculosis capitis. In the crude analysis, hairbrush sharing was significantly associated with infestation. However, after adjusting for potential confounders in sensitivity analysis, none of the included factors including maternal education, hairbrush sharing, clothes sharing, or bedroom sharing remained statistically significant. This finding suggests that head lice transmission in this population is likely influenced by a complex interplay of multiple behavioral, environmental, and socioeconomic factors, rather than being determined by any single risk factor in isolation.

The present study investigated the prevalence and associated risk factors of pediculosis capitis among primary school children, revealing an overall prevalence of 6.9%. This estimate is broadly consistent with reports from neighboring countries and comparable epidemiological settings in the Middle East. In Afghanistan, a prevalence of 8.5% has been reported among primary school children in Helmand Province, while studies from Iran have documented prevalence rates of 5.7% and 2.3% in similar school‐aged populations [30, 31, 32]. These comparable findings suggest that pediculosis capitis remains a persistent public health concern among primary school children across the region, despite variations in geographic, socioeconomic, and environmental conditions.

However, our prevalence rate is considerably lower than rates reported in other regions. Studies from neighboring Afghanistan's eastern neighbor, Pakistan, have documented exceptionally high prevalence rates of 87% and 77.4% among school children [33, 34]. These stark contrasts likely reflect differences in socioeconomic conditions, cultural practices, hygiene standards, and healthcare accessibility across regions.

The prevalence rates reported in neighboring Afghanistan and the broader Middle Eastern region shows considerable variation. For instance, the rate documented in Jordan (26.6%) and Egypt (16.7%) are substantially higher, whereas the figure reported in some regions of Iran (4.8%) is comparatively lower [35, 36, 37]. This wide disparity across the region underscores the heterogeneous nature of pediculosis transmission, which is influenced by local socioeconomic conditions, climate, population density, and cultural practices regarding hair care and personal hygiene.

Univariate logistic regression analysis revealed that only hairbrush sharing, was the primary factors examined in our model. Also, in sensitivity analysis; the result did not show the significant relationship between factors including mothers’ education, hairbrush sharing, bedroom sharing, clothes sharing and hair salon visits with pediculosis capitis. This finding aligns with the well‐established understanding that sharing personal items plays a crucial role in head lice transmission. Previous research has demonstrated that students who shared hair combs had significantly higher odds of infestation [38]. Similarly, other investigations found that children sharing combs had significantly higher infestation rates compared to those who did not share [39].

The association between maternal education and infestation in our study, though not statistically significant, is consistent with substantial evidence linking parental education to reduced risk. Research has demonstrated that children with illiterate mothers had significantly higher odds of infestation [38]. Other studies similarly found low maternal education significantly associated with higher infestation rates, with children of illiterate mothers showing higher infestation compared to those with university‐educated mothers [40]. In a comprehensive systematic review, the majority of studies demonstrated statistically higher infestation risk among children whose mothers had low education levels [11]. Educated mothers likely possess better knowledge about transmission, prevention, and treatment, enabling more effective preventive measures. The lack of statistical significance in our study may be attributed to the relatively small sample size or the uniform low educational status in the study region, limiting the ability to detect differences.

Our findings regarding household characteristics are consistent with studies identifying overcrowding as a significant risk factor. Previous research has reported that children in larger families had significantly higher odds of infestation compared to those in smaller families [39]. In refugee and displacement settings, overcrowding and communal accommodation significantly increase infectious disease transmission risks [41]. Afghanistan's prolonged conflict and patterns of displacement have contributed to living conditions characterized by overcrowding, which may facilitate the transmission of head lice and scabies [42].

Although our study did not find significant associations with hair characteristics, the literature consistently demonstrates that hair length is a critical risk factor. One study reported hair length as a significant risk factor, with girls at greater risk due to longer hair [43]. Other research found that children with long hair had significantly higher odds of infestation [39]. A systematic review revealed that a substantial number of studies demonstrated a critical impact of long hair on infestation rates [11].

The prevalence of 6.9% in our study is lower than the global pooled prevalence of 19% reported in a systematic review, with significant regional variations: Central and South America showing the highest rates, followed by Africa, Asia, North America, and Europe with the lowest [11]. This variation reflects differences in socioeconomic conditions, cultural practices, and healthcare access. Research from Iran reported an upward trend over a 4‐year period, with autumn showing higher risk than spring [29]. Such seasonal patterns may also influence transmission in Afghanistan, though our study did not examine this aspect.

The findings underscore the multifactorial nature of pediculosis capitis and the need for comprehensive interventions. Public health strategies should prioritize health education programs targeting children, parents, and teachers. Previous research found that children with poor knowledge and poor attitude had significantly higher odds of infestation [38]. Regular screening and early detection programs in schools are essential, as emphasized in the literature [44]. In settings with limited resources, simple interventions such as regular hair washing, avoiding sharing personal items, and prompt treatment can significantly reduce transmission.

## 5 Limitations

This study has several limitations that should be considered when interpreting the findings. First, the cross‐sectional design precludes the establishment of causal relationships between the investigated factors and pediculosis capitis. Second, although a wide range of demographic, socioeconomic, behavioral, and household‐related variables was evaluated, only 35 participants were diagnosed with pediculosis capitis, limiting the statistical power of the multivariable analysis and resulting in relatively wide confidence intervals. Third, the study was conducted exclusively among primary schoolchildren in Baghlan Province, which may limit the generalizability of the findings to other regions of Afghanistan with different demographic, environmental, and socioeconomic characteristics. Fourth, several exposure variables, including household practices and hygiene‐related behaviors, were obtained through questionnaire‐based self‐reporting and are therefore subject to recall and reporting bias. Fifth, because the study focused on epidemiological and behavioral determinants, other potentially relevant factors, such as molecular characterization of lice, pediculicide resistance, and environmental determinants, were not investigated and should be addressed in future studies. Sixth, diagnosis relied solely on flashlight inspection rather than fine‐tooth wet‐combing. This methodological limitation likely resulted in under‐detection of mild infestations and may have led to an underestimation of the true prevalence of pediculosis capitis. Therefore, the reported 6.9% prevalence should be considered an observed estimate rather than a definitive population prevalence.

## 6 Conclusion

This study provides one of the first epidemiological assessments of pediculosis capitis among primary schoolchildren in Baghlan Province, Afghanistan, and demonstrates that head lice infestation remains a persistent public health concern in this setting. Although several behavioral and household‐related factors showed elevated odds of infestation, none remained statistically significant in the sensitivity analysis, highlighting the multifactorial nature of pediculosis capitis and the absence of a single dominant determinant within this population. These findings underscore the need for comprehensive, school‐based prevention and control strategies that integrate routine screening, health education, early case detection, and timely treatment, alongside interventions targeting household hygiene and awareness. Strengthening such evidence‐based public health measures may substantially reduce the burden of pediculosis capitis among Afghan schoolchildren. Future adequately powered, multicenter prospective studies are warranted to further elucidate the epidemiology and determinants of infestation across different regions of Afghanistan.

## Author Contributions

**Mohammad Musawer Sharifi:** investigation. **Sayed Hussain Mosawi:** conceptualization, methodology, investigation, funding acquisition. **Ahmad Jamshid Mehrpoor:** investigation, validation, data curation. **Reza Fotouhi‐Ardakani:** investigation. **Shah Zaman Shirzad:** investigation. **Abdullah Sahar:** investigation. **Abdul Wakil Qarluq:** investigation. **Mohammad Esmail Ahmadyar:** investigation, validation. **Atefeh Khoshkchali:** formal analysis, data curation, writing – review and editing. **Hadia Azami:** validation, formal analysis, data curation. **Aida Vafae Eslahi:** methodology, software, writing – review and editing, writing – original draft, visualization. **Morteza Habibi:** conceptualization, methodology, validation. **Milad Badri:** conceptualization, methodology, software, data curation, validation, supervision, project administration, visualization; writing – original draft, writing – review and editing, resources.

## Ethics Statement

This study received ethical approval from Ghalib University (AF.GUIRB.ECBR.1403.007) and was conducted per international standards. Informed consent (written for literate, oral for illiterate participants) was obtained, and all data were anonymized.

## Conflicts of Interest

The authors declare no conflicts of interest.

## Transparency Statement

S.H.M. confirms that this manuscript provides a truthful, accurate, and transparent report of the study, with no significant omissions, and that all discrepancies from the planned study have been accounted for.

## Data Availability

All data underlying the results reported in this manuscript are available from the corresponding author (S.H.M) upon reasonable request. Due to privacy or ethical restrictions, data are not publicly accessible.
